# Machine learning framework for assessment of microbial factory performance

**DOI:** 10.1371/journal.pone.0210558

**Published:** 2019-01-15

**Authors:** Tolutola Oyetunde, Di Liu, Hector Garcia Martin, Yinjie J. Tang

**Affiliations:** 1 Department of Energy, Environmental and Chemical Engineering, Washington University, Saint Louis, Missouri, United States of America; 2 DOE Joint BioEnergy Institute, Emeryville, California, United States of America; 3 DOE Agile BioFoundry, Emeryville, California, United States of America; 4 Biological Systems and Engineering Division, Lawrence Berkeley National Lab, Berkeley, California, United States of America; 5 BCAM, Basque Center for Applied Mathematics, Bilbao, Spain; Technical University of Denmark, DENMARK

## Abstract

Metabolic models can estimate intrinsic product yields for microbial factories, but such frameworks struggle to predict cell performance (including product titer or rate) under suboptimal metabolism and complex bioprocess conditions. On the other hand, machine learning, complementary to metabolic modeling necessitates large amounts of data. Building such a database for metabolic engineering designs requires significant manpower and is prone to human errors and bias. We propose an approach to integrate data-driven methods with genome scale metabolic model for assessment of microbial bio-production (yield, titer and rate). Using engineered *E*. *coli* as an example, we manually extracted and curated a data set comprising about 1200 experimentally realized cell factories from ~100 papers. We furthermore augmented the key design features (e.g., genetic modifications and bioprocess variables) extracted from literature with additional features derived from running the genome-scale metabolic model *iML1515* simulations with constraints that match the experimental data. Then, data augmentation and ensemble learning (e.g., support vector machines, gradient boosted trees, and neural networks in a stacked regressor model) are employed to alleviate the challenges of sparse, non-standardized, and incomplete data sets, while multiple correspondence analysis/principal component analysis are used to rank influential factors on bio-production. The hybrid framework demonstrates a reasonably high cross-validation accuracy for prediction of *E*.*coli* factory performance metrics under presumed bioprocess and pathway conditions (Pearson correlation coefficients between 0.8 and 0.93 on new data not seen by the model).

## Introduction

Despite the rapid advances in designing synthetic biological systems for various important applications, prediction of cellular behavior remains a challenge [1]. High fidelity predictive tools are critical for enabling rational strain design. While earlier tools were based on steady-state constraint-based methods, newer tools leveraging kinetic information [2] and integrating omics data [3] have been developed to improve model prediction accuracy. However, the practical utility of these tools has not been extensively demonstrated, and the majority of metabolic engineering efforts are still currently based on experience, intuition, and laborious testing of large numbers of designs. This is because mechanistic models cannot account for complete bioprocess variables or metabolic regulatory interactions, while hidden physiological constraints (such as metabolite channeling, metabolic burdens, strain stability, changes in enzyme expression in different phases of cell growth, and strain nongenetic variations) lead to suboptimal cell metabolisms [4,5]. Quantitative modeling of these phenomena is critical for the success of metabolic engineering designs. Since mechanistic models may not be comprehensive enough to guarantee accurate predictions, data-driven approaches have shown promise for accounting for nontrivial factors without detailed knowledge of cellular processes [6]. Given the extensive microbial researches to produce variety of bio-products, there has been a lot of interests in utilizing published metabolic engineering data to facilitate new designs and shorten the ‘design-build-test-learn’ paradigm of strain improvement [7]. Currently, metabolic engineering case studies are rapidly growing. Databases for strain development and related omics studies are being developed [1,8–13]. These databases provide genomic information to gain insights into cellular processes and their regulations. On the other hand, there are still few knowledge engineering efforts to extract and standardize holistic bioinformatics from the published papers including genetic modification strategies, cell physiological responses, and bioprocess conditions. In fact, these published papers may contain wealthy resources and lessons to support machine learning for strain designs, and thus leveraging published data may assist metabolic models to predict accurate cell performances and tradeoffs among TRY (titer, rate and yield) under realistic conditions (e.g., product inhibitions and suboptimal pathway functions, etc.).

**Fig 1:**
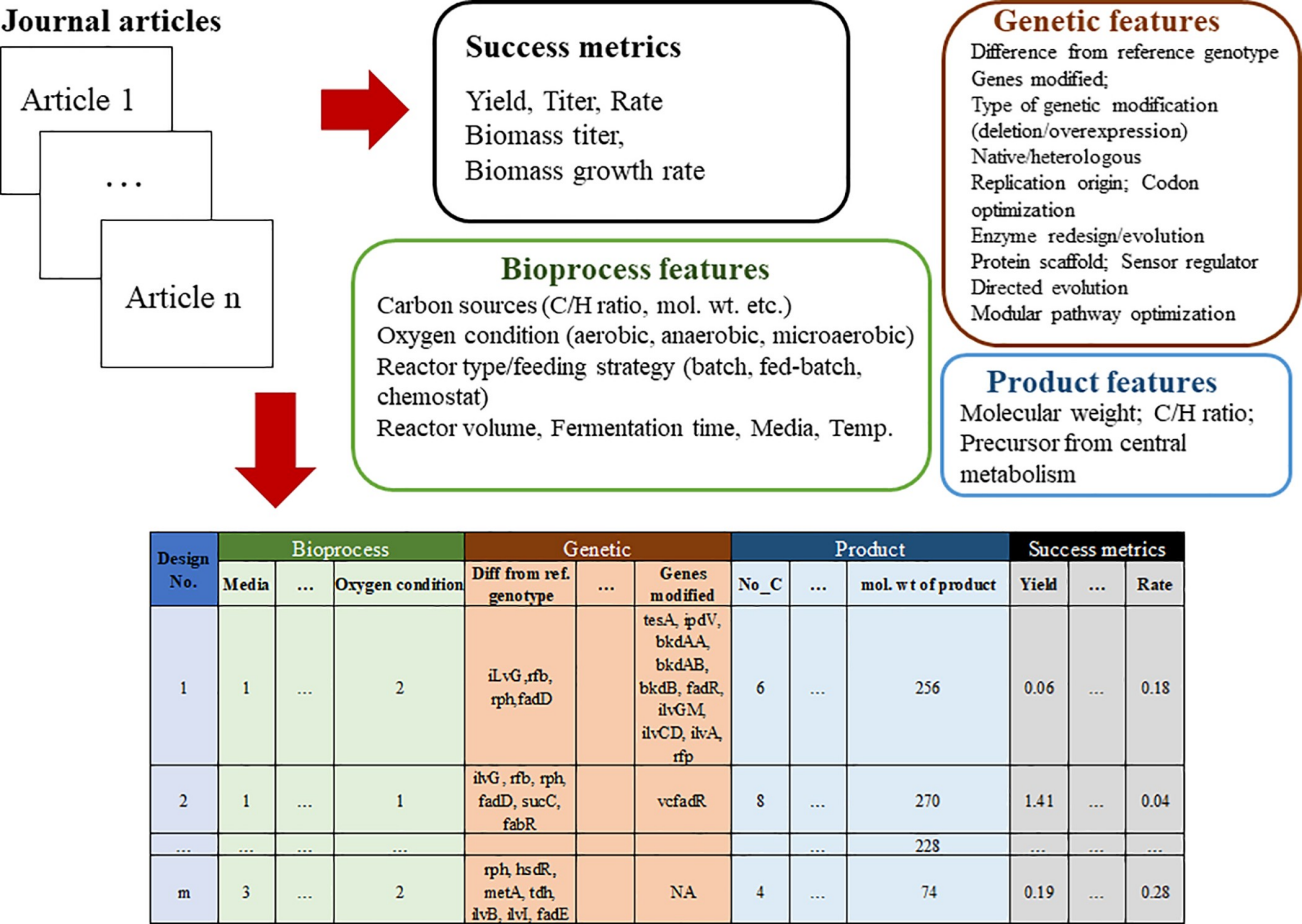
Database curation and feature extraction methodology.

**Fig 2:**
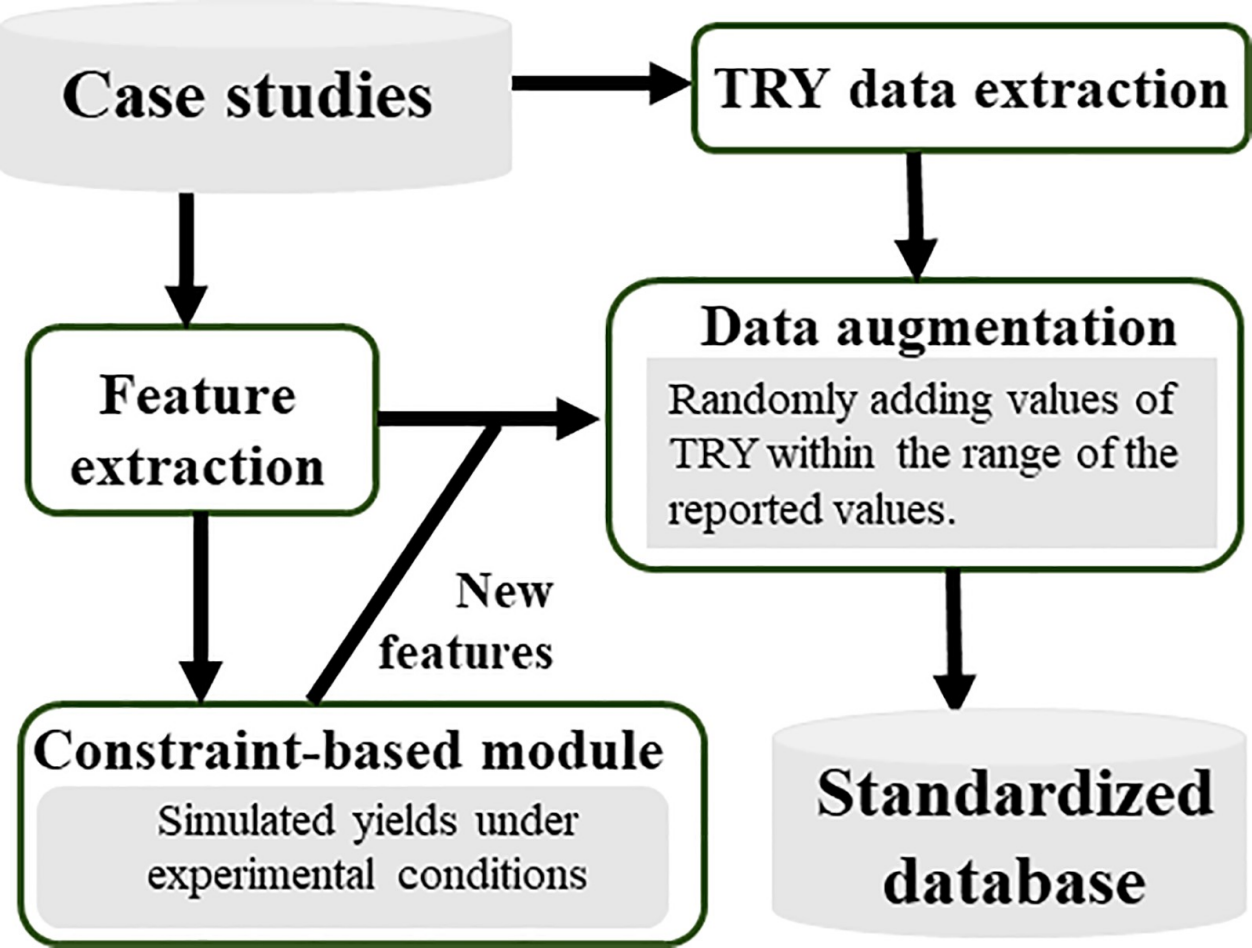
Feature additions via genome scale model simulations and data augmentation based on case studies described in the literatures.

Nevertheless, the use of literature data for computer based strain design and performance predictions still faces difficulties: 1) Lack of standardization of data reports from different research labs, 2) Incomplete production metrics (titer, yield, and rate) and experimental parameters; 3) Sparse data coverage (most of the available data are focused on a few popular products and designs). Moreover, it has been demonstrated that machine learning models with high predictive fidelity may not be useful to provide mechanistic explanations [14].To digest the noisy information from thousands of metabolic engineering publications, data collections, curations, and feature categorizations must be performed to make sufficiently large data sets assessable to machine learning tools. Such knowledge engineering requires an extremely large amount of manpower. To resolve this problem, this proof-of-concept study has manually extracted data from ~100 published *E*. *coli* biomanufacturing papers over the past decade (Fig 1). Advanced machine learning techniques (data augmentation, ensemble learning) are employed to alleviate the challenges of sparse and small data sets. Constraint-based modeling is used to provide additional features for training the ensemble machine learning models (Fig 2). The hybrid platform provides reasonable estimations of *E*.*coli* TRY performance, which may open a new direction for metabolic modeling and strain design.

## Results and discussion

### Description of curated database

**Fig 3:**
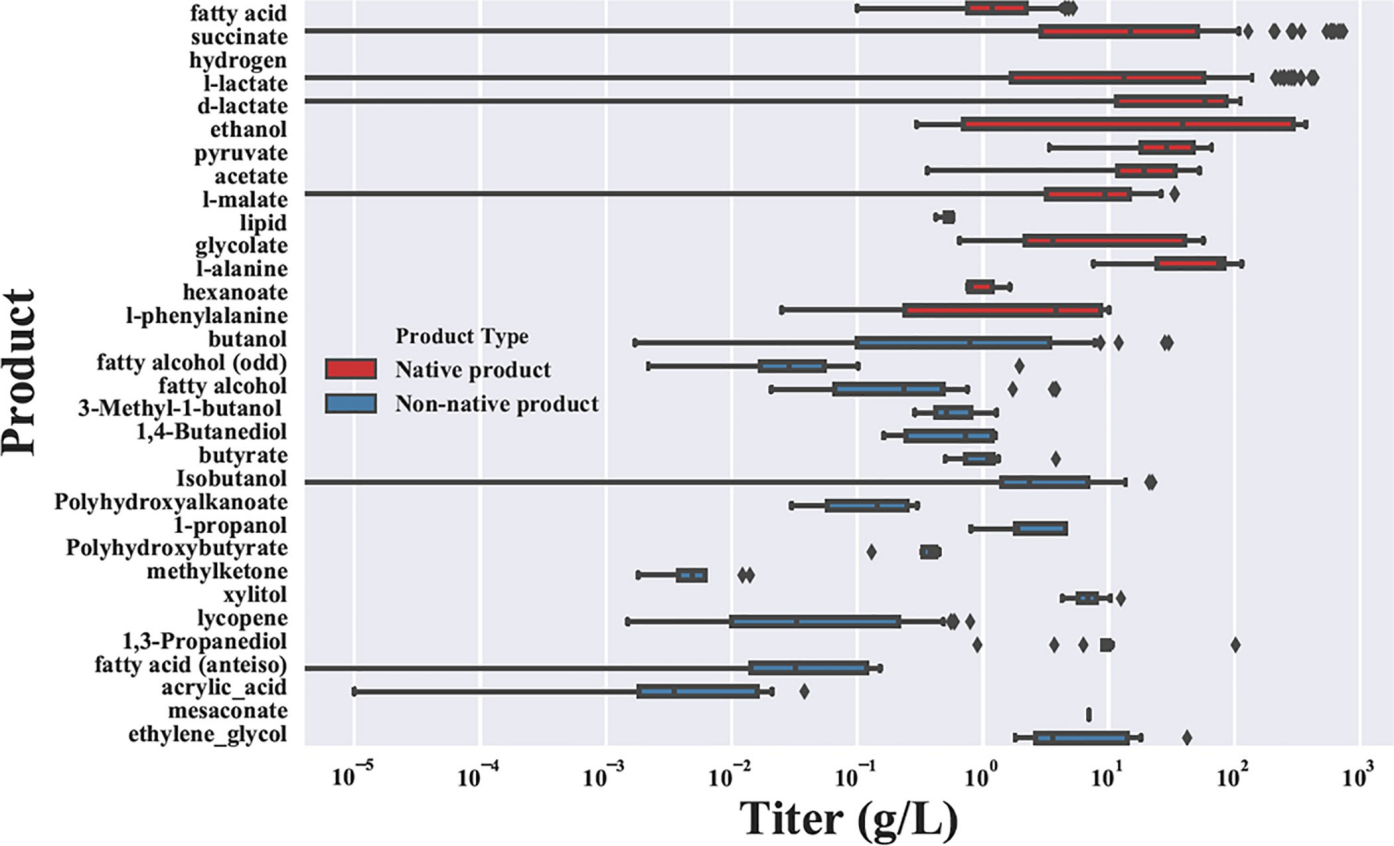
Summary of curated database showing distribution of titers (units in g/L) for 25 different products from the bacterium *E*. *coli*.

This study focuses on *E*. *coli* platforms with native or heterologous pathways for producing small molecules. About 1200 metabolic engineering designs for producing more than 20 compounds were manually extracted and estimated from ~100 journal articles (provided in the supplementary excel file) to the authors' best efforts. The genetic strategies and microbial fermentation conditions were extracted based on Table 1, as proposed by the previous paper [15,16]. In brief, data are organized as six categories, including carbon sources, bioprocess conditions (e.g., medium types), genetic modification strategies, product features (e.g., molecular weight, enzyme steps from central pathways, etc.), production metrics TRY, and other unaccountable factors. To summarize extracted data, the distribution of titer (the most commonly reported metric) for the different compounds is shown in Fig 3, where native products (synthesized by native enzymes in *E*.*coli*) often have higher titer than non-native products (synthesis via heterologous pathways).

**Table 1:** Metabolic engineering design factors template used for feature extraction. Sample values are taken from [19]. Features that refer to a list of genes are entered as a vector of ones and zeros as categorical numbers. For example, in the sample values, ‘het_gene’ (whether the gene inserted/overexpressed was heterologous) is entered as 1,0,0 meaning alsS is heterologous while ilvC, ilvD are not. YE stands for yeast extract.

		Feature	Description	Sample value
**carbon source characterization**	1	cs1	first carbon source	1
	2	cs1_mw	first carbon source molecular weight	180
	3	cs_conc1	first carbon source concentration (mM)	111
	4	CS_C1	mol C in first carbon source	6
	5	CS_H1	mol H in first carbon source	12
	6	CS_O1	mol O in first carbon source	6
**Bioprocess conditions**	7	reactor_type	type of reactor (continuous, batch or fed-batch)	1
	8	rxt_volume	working volume of reactor (L)	2
	9	media	media used for fermentation (M9,AM1,AM2, M9+ yeast extract,LB,NBS,TB,other rich media)	YE
	10	temp	temperature of medium used for fermentation (oC)	37
	11	time	total time for fermentation	36
**Genetic modifications**	12	oxygen	oxygen condition in reactor (aerobic, anaerobic, microaerobic,extra aerobic)	2
	13	sbg_ref	reference strain in the study	BFA7.001(DE3) PCT01
	14	s_ref_gen	genes modified from the strain MG1655	lacI, rrnB, lacZ, hsdR514, araBAD, rhaBAD, zwf, mdh, frdA, ndh, pta, poxB, ldhA,T7 RNA polymerase
	15	s_gen_mod	type of gene modification: insertion/deletion	0,0,0,0,0,0,0,0,0,0,0,0,0,1
	16	gene_mod	genes modified from reference strain of study	alsS, ilvC, ilvD
	17	gene_del	whether or not the gene was deleted	0,0,0
	18	gene_ovr	whether or not the gene was overexpressed	1,1,1
	19	het_gene	is the gene heterologous? (yes/no)	1,0,0
	20	rep_origin	plasmid copy numbers	5,5,5
	21	codon_opt	codon optimization?	0,0,0
	22	sen_reg	sensor regulator?	0,0,0
	23	enz_design	enzyme redesign evolution?	0,0,0
	24	protein_scaffold	protein scaffolding?	0,0,0
	25	dir_evo	direction evolution?	0
	26	Mod_path_opt	modular pathway optimization?	0
**Product characterization**	27	prod_name	name of the product	Isobutanol
	28	no_C	mol C in product	4
	29	no_H	mol H in product	10
	30	no_O	mol O in product	1
	31	no_N	mol N in product	0
	32	mw	molecular weight of product	74
	33	precursor	precursor from central metabolism	6
	34	enz_steps	number of enzyme steps from precursor	5
	35	atp_cost	number of atp molecules needed from precursor to product	0
	36	na_cost	number of nadh/nadph molecules needed from precursor to product	2
**Production metrics**	37	yield_1	yield in gProduct/g Carbon source fed	0.0405
	38	yield_2	yield in gProduct/g Carbon source consumed	NA
	39	yield_3	yield in gProduct/g Biomass	0.623
	40	titer	concentration of product in g/L	0.81
	41	rate	maximum productivity in g Product/ L /h	0.0225
	42	bio_titre	biomass concentration (g/L)	1.3
	43	bio_grw_rate	biomass growth rate in exponential phase (/h)	0.45
**other**	44	gen_info	are all the genetic modifications in the paper fully captured by the above categories? (yes/no)	1
	45	env_info	are all the reactor conditions in the paper fully captured by the above categories? (yes/no)	1

**Fig 4:**
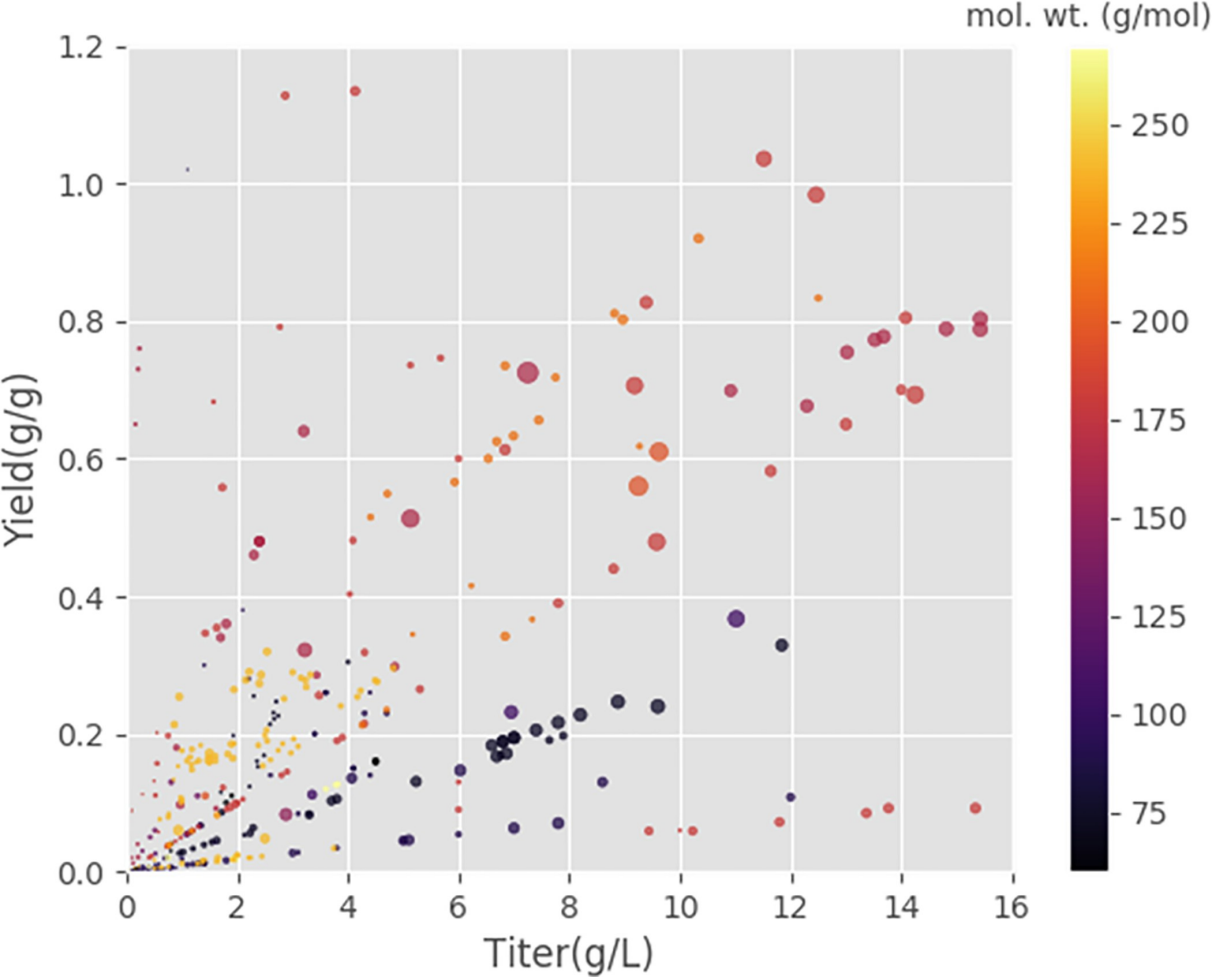
Comparison of production metrics (titer, rate, and yield). The size of the dots corresponds to the rate values (in g/L/h scaled by the minimum and maximum value– 0.000043 and 10.83 g/L/h respectively). Molecular weight of each product (g/mol) is shown by the color gradient of the dots (color bar).

Biomanufacturing requires cell factories to achieve the desired TRY. Fig 4 provides correlations among the three metrics as well as product molecular weight (mol. wt). There appears to be positive correlations between titer and yield (i.e., an increase of feedstock conversion improves product concentration). However, production rate can be impaired by very high production yield/titer (i.e., elevation of yield reduces carbon resources to generate ATP and biomass for cell well-being, while the high titer may stress cell physiologies). In general, it is difficult to maximize all three biomanufacturing metrics due to the tradeoff of carbon/energy metabolisms and product inhibitions. Fig 4 shows that these maximal production rates from published case studies are in the medium ranges of titer (6~10g/L) and yield (0.45g/g~0.75g/g), while some products (e.g., succinate) achieve very high yields (>1g /g substrate) due to cellular carbon fixations. These extracted data sets can be used as the base for machine learning to predict fermentation performance and tradeoffs.

### Identification of critical metabolic engineering factors

**Fig 5:**
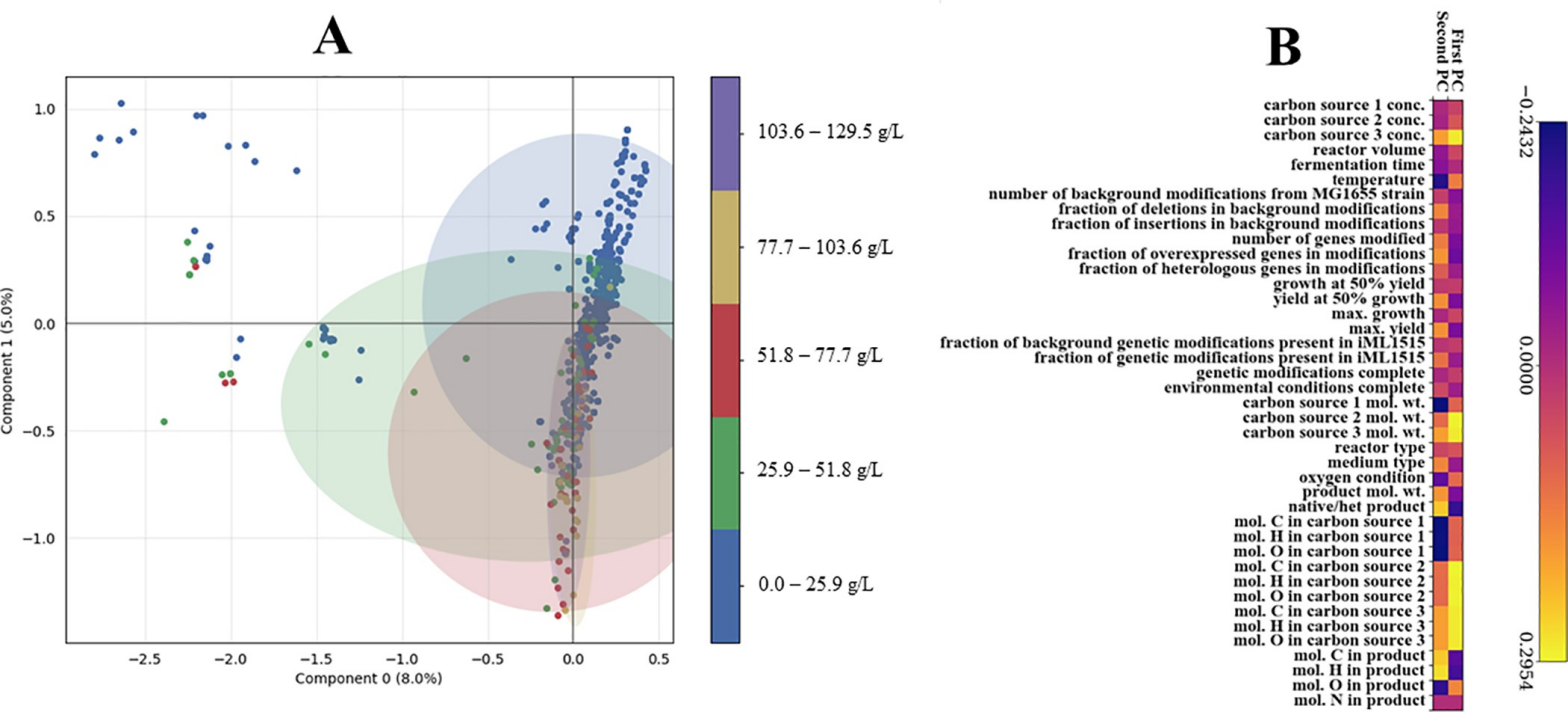
Inferring possible influential factors on metabolic engineering design performance. A. First two principal components from multiple correspondence analysis (MCA). The labels correspond to titer values in g/L. The shaded areas for each point show the predicted area within which all points have a high probability of belonging to the specified titer range. B. Impact of different influential factors on first two principal components from principal component analysis (PCA). PCA plot shown in S1 Fig in S2 File. Carbon source 1, 2 and 3 are used to capture the cases in which more than one carbon source was used. If only one was used, corresponding entries of carbon source 2 and 3 were set to zero. *E*.*coli* MG1655 was taken as the reference strain and all modifications done to get the background strain used in each study were captured as ‘background modifications’. The scores describe the relative contribution of each feature to the principal components.

Many factors may play a role in optimal metabolic engineering design. To analyze the data based on our custom-designed features, we utilized the complementary approaches of multiple correspondence analysis (MCA) [17] and principal component analysis (PCA) [18]. MCA is more suited for categorical data while PCA works best with continuous data. Interestingly, both techniques yielded similar results (clustering of the high titer values around the zero of the first principal component and along the second principal component). Fig 5A shows the plot of the first two principal components of the MCA with the titer values superimposed. Regions of high titers are clustered along the second principal component and most have a value of zero for the first principal component. This indicates that the factors that make up the second principal component are critical for high titers. The contributions of different factors to the first two principal components of the PCA are shown in Fig 5B and are indicative of their relative influence on microbial cell performance. Bioprocess factors such as reactor volume, temperature, oxygen conditions (anaerobic or aerobic), medium types, substrate characteristics (molecular weight, C, H, O composition) have an impact on cell performance. Therefore, further categorization and addition of bioprocess conditions as model inputs can improve machine learning accuracy. On the other hand, outcomes from genetic factors/modifications are more uncertain due to complex genomic causes and metabolic responses to engineered pathways. To overcome this problem, the *E*. *coli* genome-scale metabolic network reconstruction (iML1515) is simulated to estimate metabolic network capabilities (subject to the experimental genetic modifications and bioprocess conditions) (Eqs 1–5). The results of the simulations are used as additional features for training the machine learning models. The hybrid of constraint-based simulation with machine learning provides a more realistic estimation of cell performance.

### Model performance validation

**Fig 6:**
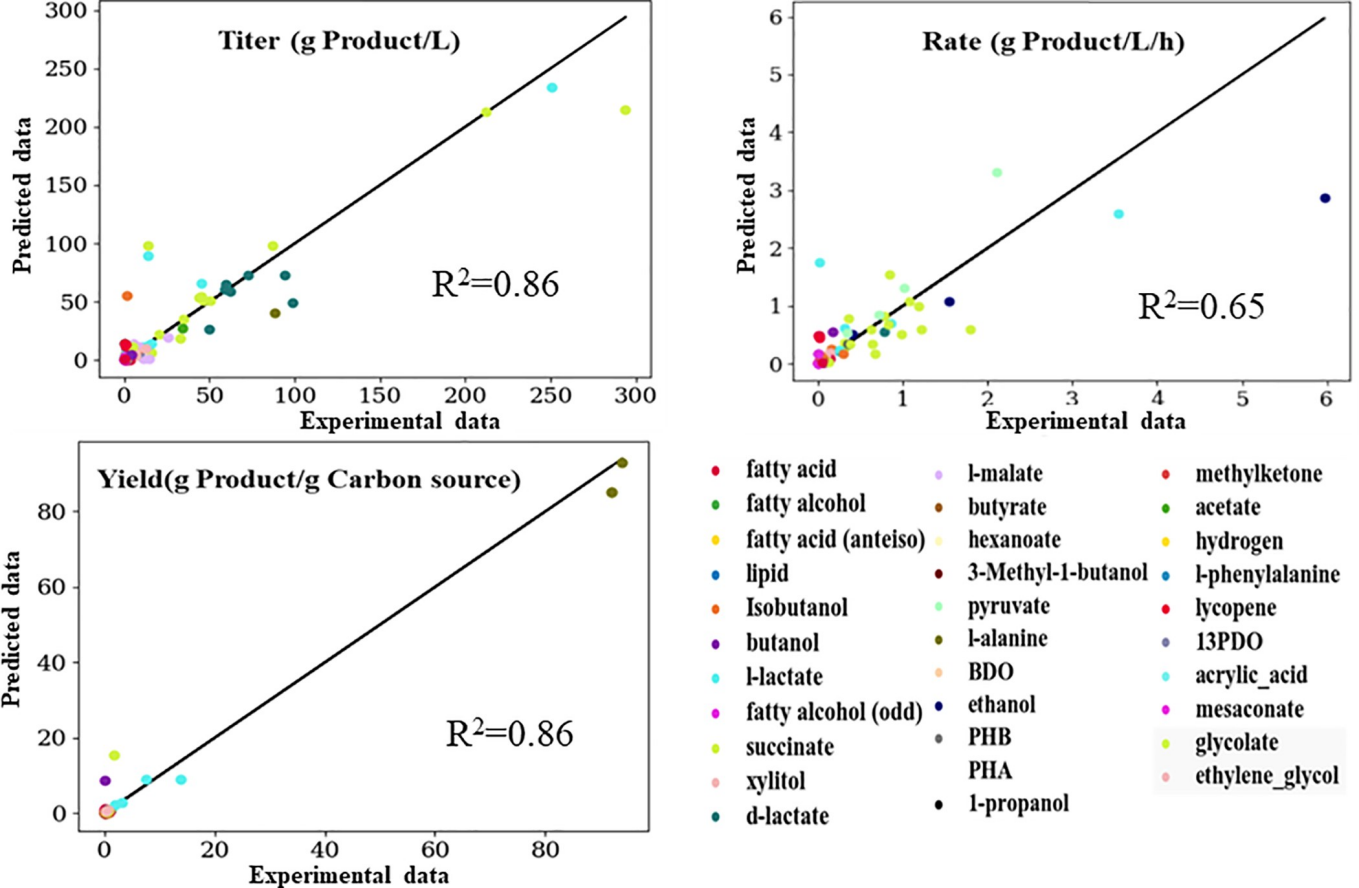
Prediction of production metrics TRY. R^2^: coefficient of determination. Solid lines are shown on the diagonal that represent where all the points would fall for perfect prediction. A scaled version of Fig 6 is presented in S4 Fig in S2 File (enabling the fit to visualized without the outlier effects). The data points are scaled based on the maximum value (titer, rate or yield) for the particular product in our curated database.

**Fig 7:**
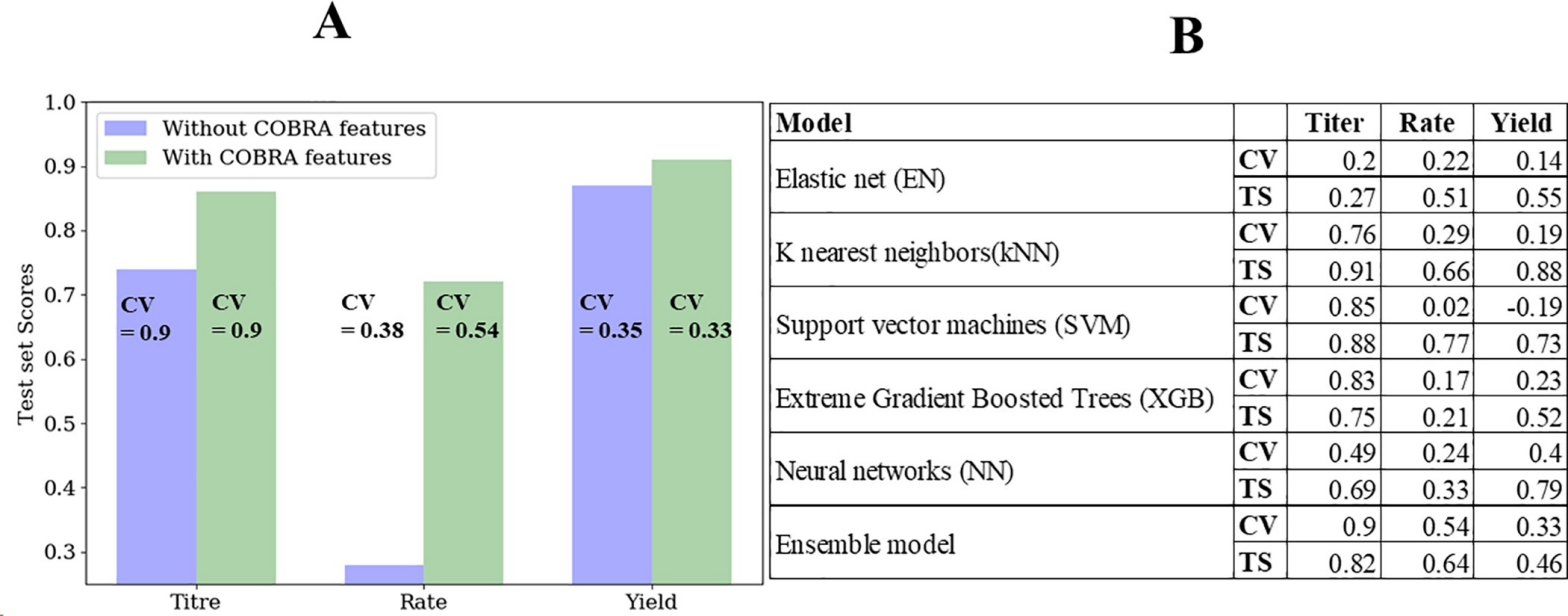
Model performance analyses. A. Quantification of the effect of COBRA (Constraint-Based Reconstruction and Analysis)—based features on model performance. CV stands for the best cross validation accuracy (R^2^ values). Higher scores imply a better fit. B. Comparing individual machine learning performance with ensemble model. TS stands for Test Scores (R^2^ values). CV stands for the best cross validation accuracy (R^2^ values). Higher scores imply a better fit.

The predictive ability of the machine learning model on the test data set (not previously seen by the model) is shown Fig 6. Despite the small data set size (~1200) from a variety of studies (~120), the predictive performance of the model is high for native and non-native *E*. *coli* products. The use of techniques such as data augmentation and stacked regression (discussed in the methods section) significantly improve model performance. The model also does well for products with wide ranges of titer, rate, or yield values (for example, L-lactate and succinate). The use of extra features from constraint-based simulations as well as ensemble learning of different machine learning models improves predictive performance (Fig 7). Some models (like Extreme Gradient boosted trees, which is itself an ensemble technique) give good performance for one metric but not others. Others, like Support Vector Machines (SVMs), give high test scores but the cross-validation accuracies are not robust, showing the model might not generalize well to new data not seen by the model. The final model (stacked regressor) gives a balanced performance across all metrics TRY. One important thing to note is that most of the experimental data points are clustered at very low and very high values with very few points in the middle. Thus, the trained model will differentiate between very good and relatively poor performing strains but might struggle with average performing strains. Obtaining more experimental data with average performance will enable more robust model predictions. We experimented with scaling the yield data with the maximum computed theoretical yield for each product to enable a fairer comparison across products. However, the performance of the model on the scaled data did not improve with this scaling.

### Model improvement

**Fig 8:**
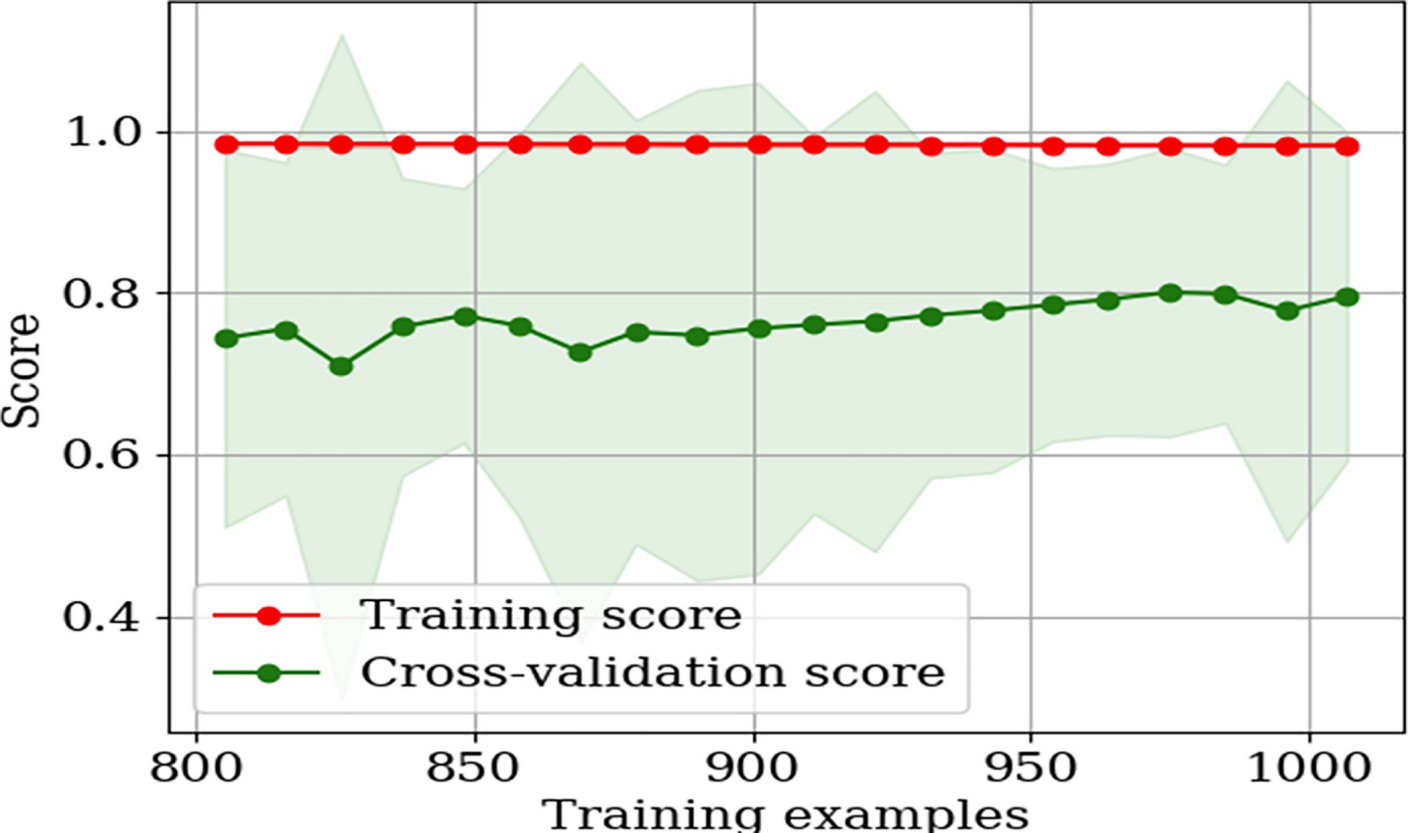
Titer learning curve as the function of size of training data set. The training scores (R^2^) and cross validation (CV) scores (also R^2^) are shown. Below 800 training examples, the cross-validation accuracies variation were too large. The hybrid model can fit the training data set (red points) well irrespective of the number of training examples. The cross-validation scores improve slightly with more data points. This implies that more feature engineering (and not necessarily more data) would be necessary to significantly improve model performance.

While there is a decent correlation between experiment and model predictions, cross validation analyses reveal variability in model predictions. There are three limitations for machine learning approaches. First, data extraction and curation from published data are prohibitively time-consuming. This is because metabolic engineering papers do not have standard reports of yield/titer and cell productivity can be strikingly different under different growth stages. Manual estimation of production metrics from incomplete published data sets is bound to contain human subjective errors. Second, fermentation media are often undefined (with significant amount of yeast extract or other secondary substrates), which makes yield calculations inaccurate (i.e., the model predictions on production rate and yield are subpar to titer). Third, our data size and extracted features are still limited, and there are other influential factors (such as waste byproduct secretion during fermentation and strain stability) that are ignored during data curations. Therefore, high-accuracy computational methods for predicting complex cellular phenomena under bioprocess conditions remain challenging. Much effort and resources must be devoted to data curation, feature extractions, and tailoring of machine learning techniques for application to metabolic engineering data. For example, learning curves demonstrate the possibility of more robust model predictions with larger data sets (Fig 8). Learning curves for yield and rate are shown in S2 and S3 Figs in S2 File.

## Methods

### Database curation

*E*. *coli* is the most common platform for metabolic engineering. The database is manually curated from metabolic engineering literature on the production of diverse chemicals from *E*. *coli* grown on different substrates. The feature selections and data curation strategy are based on our previous work [15]. This involves identifying possible influential factors a priori (shown in Fig 1 and Table 1). The full list of papers is shown in the supplementary file. A sample of feature extraction from a journal paper is shown in Table 1. The list of features is iteratively updated based on model performance. Because of incomplete experimental descriptions found in some papers, comprehensive data extraction may be difficult. Two additional features are used to describe whether or not all the genetic and experimental conditions have been fully included by the feature list.

### Constraint-based simulations

**Fig 9:**
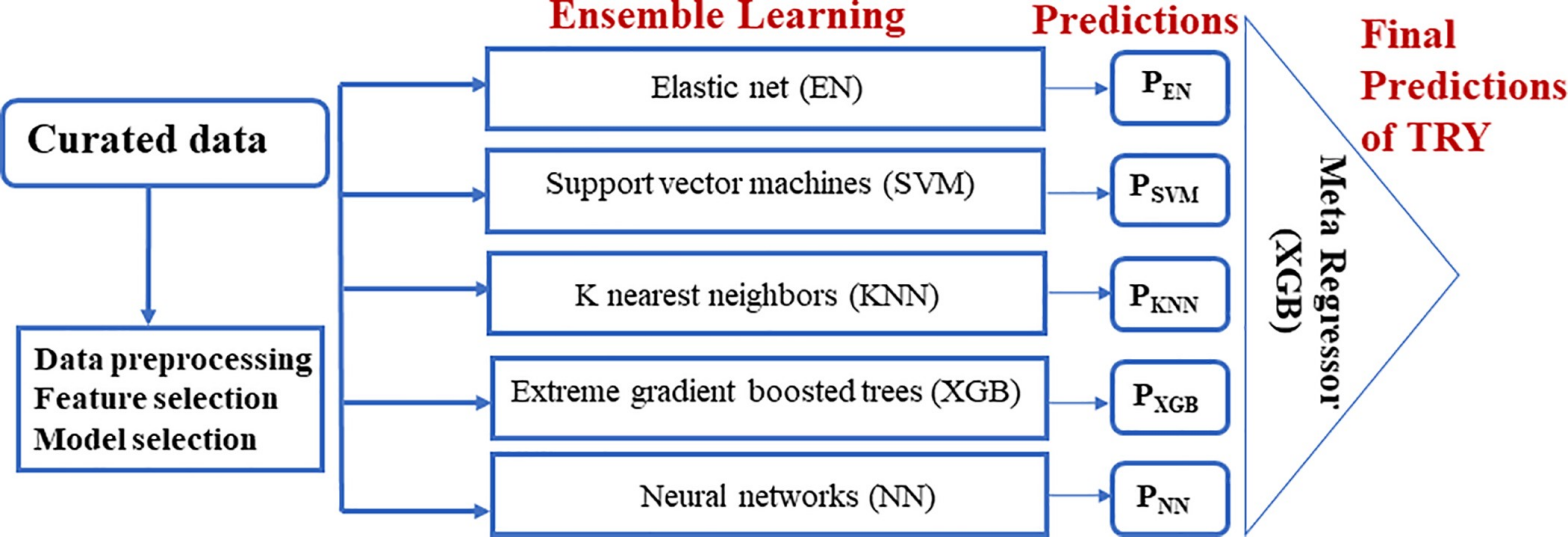
Machine learning pipeline. Ensemble learning using stacked regressors.

Given the genetic and environmental background, the most recent *E*. *coli* genome-scale metabolic reconstruction, iML1515 [20] is used to simulate theoretical microbial yields based on reaction stoichiometry. First, iML1515 flux network is modified based on each case study (e.g., gene knockouts), while inflow and outflow fluxes are constrained based on bioprocess conditions (such as carbon sources, aeration level in the reactor, growth rate, etc.) by setting the upper and lower bounds of the associated reactions to zero. A flux balance analysis (FBA) simulation (maximize biomass growth objective) is then performed to test if the resulting model is feasible. Then, further genetic interventions (in form of knockouts or overexpression) are similarly simulated so that the *in-silico* model represents the actual experimental conditions as closely as possible (Eq 1). To simulate overexpression of a biosynthesis pathway, the lower boundary of the associated flux is set to 10% of the theoretical maximum flux through this pathway. To characterize the metabolic capacity of the network after genetic modification under the applied process conditions (feature engineering), we have computed the product and biomass yield under different constraints. These are: maximum biomass growth and product yield, maximum biomass growth at 50% maximum product yield, maximum product yield at 50% biomass growth (Eqs 2–5). FBA results are used as additional features used in training the various machine learning models employed, which captures the metabolic network capabilities (in terms of feature variables) for data driven models. For certain cases, the iML1515 model (with the experimental genetic and bioprocess conditions imposed) can predict feasible solution spaces. The corresponding FBA can be constrained based on biomass growth, the number of genes modified, and the fraction of those genes that are overexpressed or deleted. The FBA simulation outcomes (simulated yields under presumed experimental conditions) are fed into machine learning pipelines as additional features from Table 1 for model training (Figs 2 and 9).

$$\begin{array}{l} \max\;c_{b}v\\ subject\;to\;\left\{ \begin{array}{c} S.v=0\\ {lb}_{j}^{e}\leq v_{j}\leq {ub}_{j}^{e} \end{array}\right. \end{array}$$ (1)

*where c_b_ is a vector of zeros with one for the biomass flux variable* ${lb}_{j}^{e}$ *and* ${ub}_{j}^{e}$ *are the flux bounds adjusted based on the bioprocess condtions and genetic modifications* (*using the gene–to–protein relationships*)

$$\begin{array}{l} \max\;c_{p}v\\ subject\;to\;\left\{ \begin{array}{c} S.v=0\\ {lb}_{j}^{e}\leq v_{j}\leq {ub}_{j}^{e} \end{array}\right. \end{array}$$ (2)

*where c_p_ is a vector of zeros with one for the desired product flux variable*

$$\begin{array}{l} \max\;c_{b}v\\ subject\;to\;\left\{ \begin{array}{c} S.v\;=\;0\\ {lb}_{j}^{e}\leq v_{j}\leq {ub}_{j}^{e}\\ c_{p}v=0.5v_{p}^{*} \end{array}\right. \end{array}$$ (3)

*where* $v_{p}^{*}$ *is a maximum product flux computed by* *Eq* 2

$$\begin{array}{l} \max\;c_{p}v\\ subject\;to\;\left\{ \begin{array}{c} S.v\;=\;0\\ {lb}_{j}^{e}\leq v_{j}\leq {ub}_{j}^{e}\\ c_{b}v=0.5v_{b}^{*} \end{array}\right. \end{array}$$ (4)

*where* $v_{b}^{*}$ *is a maximum product flux computed by* *Eq* 1

$$y_{b}^{max}\;=\;\frac{v_{b}^{*}}{v_{c}^{*}},\;y_{p}^{max}\;=\;\frac{v_{p}^{*}}{v_{c}^{p}}\;,y_{b}^{50p}\;=\;\frac{v_{b}^{50p}}{v_{c}^{50p}}\;,y_{p}^{50b}\;=\;\frac{v_{p}^{50b}}{v_{c}^{50b}}$$ (5)

*where* $v_{c}^{*},v_{c}^{p},\;v_{c}^{50p},v_{c}^{50b}$ *are carbon source uptake rates from Eqs* 1–4 *respectively*

$v_{p}^{*},v_{p}^{50b}$ *are the product fluxes from Eqs* 2 *and* 4 *respectively*

${\;v}_{b}^{*},v_{b}^{50p}$ *are the biomass growth rates from Eqs* 1 *and* 4 *respectively*

$y_{b}^{max}$ *is the maximum biomass yield*

$y_{b}^{50p}$ *is the biomass yield at* 50% *of the maximum product flux*

$y_{p}^{max}$ *is the maximum product yield*

$y_{p}^{50b}$ *is the product yield at* 50% *of the maximum biomass growth rate*

### Data pre-processing and augmentation

Principal component analysis and data standardization (using mean and standard deviation) are used to transform the input data (The first 40 components of the PCA are used in training the model). The data set is divided into training, validation, and test sets (test set is 10% of the whole data set). The test set is handled separately to prevent the data leakage (where some properties of the test distribution are inadvertently used in tune the model resulting in overly optimistic prediction accuracies). For the training and validation sets, data augmentation (a popular technique used in computer vision) [21] was employed as follows: for each data the point, n number of points where generated by randomly adjusting the values of titer, rate and yield within t % of the reported value. A grid search is used to tune hyperparameters *n* and *t*. *n* ranged from 10 to 90 and *t* ranged from 0.1% to 1%. Final values of *n* and *t* used are 50 and 0.1% respectively. Data augmentation improved the cross validation and test set accuracies.

### Ensemble learning and hyperparameter tuning

An overview of the machine learning pipeline is shown in Fig 9. Different machine learning models are tested. Support vector machines, elastic nets, random forest, gradient boosted trees, k nearest neighbors, and neural network models (densely connected, 5 hidden layers (100 neurons each) with batch normalization and dropout between layers) are trained separately on the training set. The results (test scores, cross validation and learning curves) of each of the ML models are shown in the supplementary file. Ensemble learning is then performed using the output of the different ML models. This is done with a stacked regressor (using gradient boosted trees as a meta regressor). This helps to combine the best effects of the different machine learning models to obtain higher predictive accuracies. Hyper parameter tuning for each machine learning model and final stacked regressor was based on grid search with five-fold cross validation. The modeling framework was implemented in Python. Scikit-learn [22], XGBoost [23] and Keras [24] machine learning libraries were used in the supervised learning module. COBRApy [25] implementations of constraint-based methods were used. Visualizations generated with Matplotlib [26] and Bokeh (http://bokeh.pydata.org) libraries.

## Supporting information

S1 File Excel file containing list of journal papers and link to data extracted. (XLSX)

S2 File Supplementary figures describing the results. **S1 Fig. First two principal components from principal correspondence analysis (PCA).** Color labels correspond to increasing titer values (1 being lowest and 4 being highest). **S2 Fig. Rate learning curve. S3 Fig. Yield learning curve. S4 Fig. Prediction of production metrics (titer, yield and rate).** The yield, titer and rate are scaled by the maximum reported values for each product in our curated database.
(DOCX)
