# Supplementary material for: Machine learning framework for assessment of microbial factory performance
Source: PLoS One. 2019 Jan 15;14(1):e0210558. doi: 10.1371/journal.pone.0210558 (PMC6333410; doi:10.1371/journal.pone.0210558)
Supplement: S2 File — S1 Fig. First two principal components from principal correspondence analysis (PCA). Color labels correspond to increasing titer values (1 being lowest and 4 being highest). S2 Fig. Rate learning curve. S3 Fig. Yield learning curve. S4 Fig. Prediction of production metrics (titer, yield and rate). The yield, titer and rate are scaled by the maximum reported values for each product in our curated database. (DOCX) [file pone.0210558.s002.docx]

**Supplementary file for:**

**Machine learning framework for assessment of microbial cell factory performance**

Tolutola Oyetunde^1^, Di Liu^1^, Hector Garcia Martin^2,3,4,5^ and Yinjie J. Tang^1^*

^1^Department of Energy, Environmental and Chemical Engineering, Washington University, Saint Louis, Missouri, 63130.

^2^ DOE Joint BioEnergy Institute, Emeryville, California 94608, USA

^3^ DOE Agile BioFoundry, Emeryville, California 94608, USA

^4^ Biological Systems and Engineering Division, Lawrence Berkeley National Lab, Berkeley, California 94720, USA

^5^ BCAM, Basque Center for Applied Mathematics, Bilbao, Spain

*Corresponding author, [yinjie.tang@seas.wustl.edu](mailto:yinjie.tang@seas.wustl.edu)


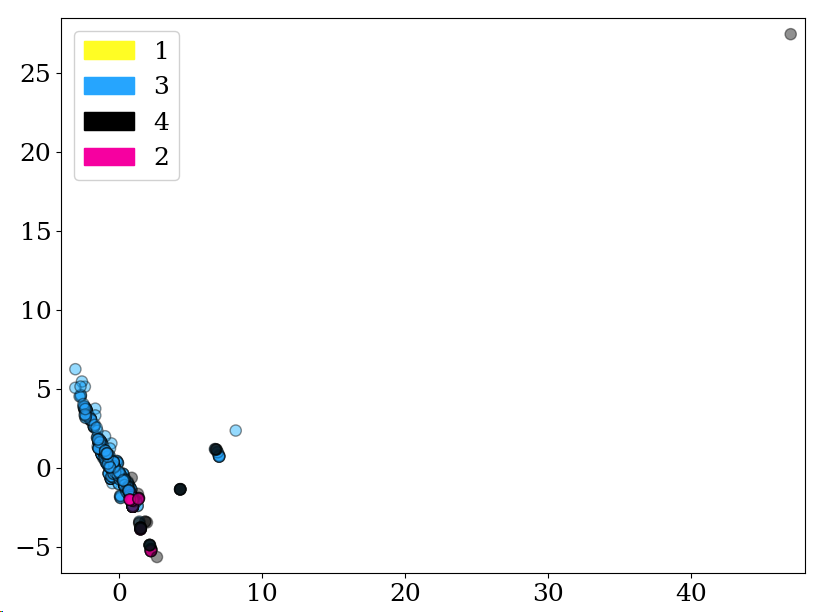


**S1 Fig. First two principal components from principal correspondence analysis (PCA).** Color labels correspond to increasing titer values (1 being lowest and 4 being highest).


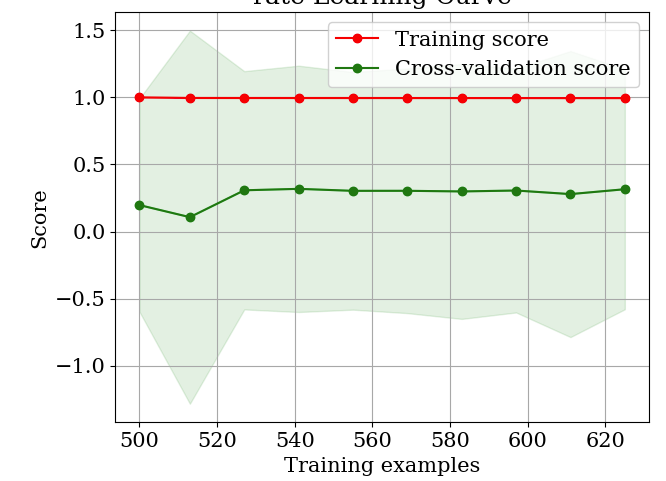


**S2 Fig. Rate learning curve**


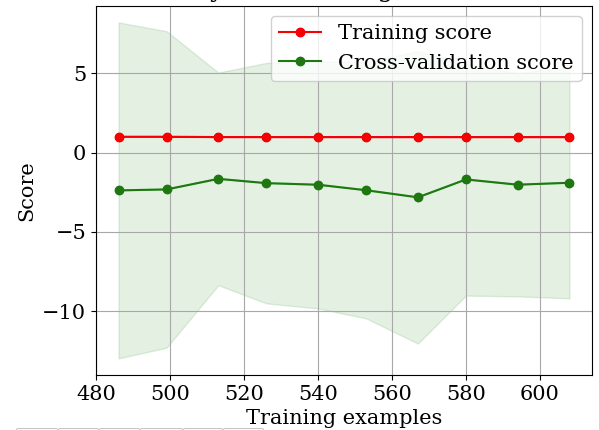


**S3 Fig. Yield learning curve**

**
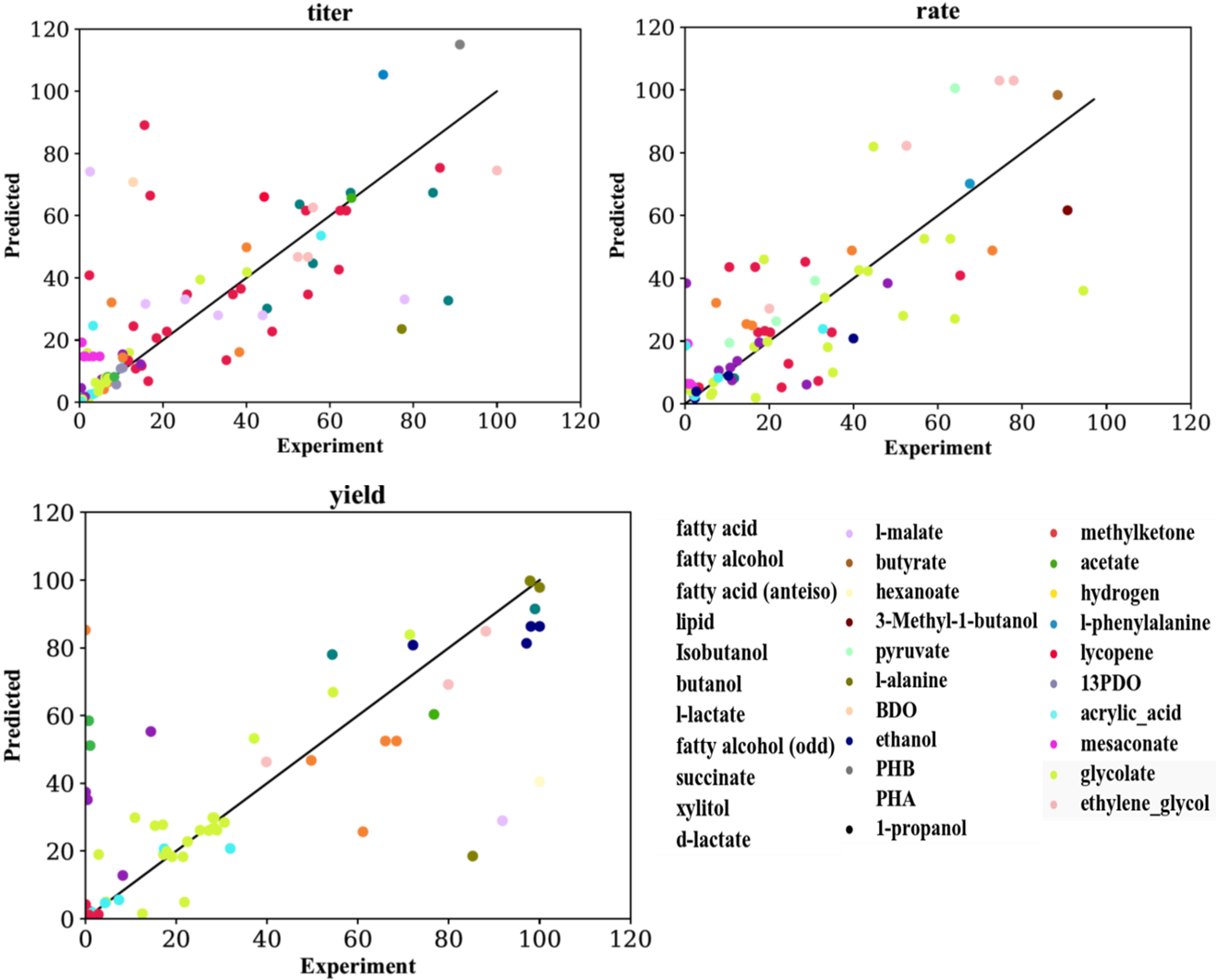
**

**S4 Fig. Prediction of production metrics (titer, yield and rate).** The yield, titer and rate are scaled by the maximum reported values for each product in our curated database
